# Novel Experimental In-Office Bleaching Gels Containing Co-Doped Titanium Dioxide Nanoparticles

**DOI:** 10.3390/nano12172995

**Published:** 2022-08-30

**Authors:** Matheus Kury, Rochelle D. Hiers, Yan D. Zhao, Mayara Z. D. Picolo, Jessica Hsieh, Sharukh S. Khajotia, Fernando L. Esteban Florez, Vanessa Cavalli

**Affiliations:** 1 Division of Operative Dentistry, Department of Restorative Dentistry, Piracicaba School of Dentistry, University of Campinas, Piracicaba 13414-903, SP, Brazil; 2 Division of Dental Biomaterials, Department of Restorative Sciences, College of Dentistry, University of Oklahoma Health Sciences Center, Oklahoma City, OK 73117, USA; 3 Department of Biostatistics and Epidemiology, Hudson College of Public Health, University of Oklahoma Health Sciences Center, Oklahoma City, OK 73104, USA

**Keywords:** tooth bleaching, hydrogen peroxide, light irradiation, nanoparticles

## Abstract

The present study reports on the development and testing of novel bleaching agents containing co-doped metaloxide nanoparticles (NP; 0%, 5%, 10% *v/w*) and hydrogen peroxide (HP, 0%, 6%, 15%, and 35%). Bovine blocks (n = 200, A = 36 mm^2^) were obtained and randomly distributed into experimental groups (n = 10/group). NPs were incorporated into gels before bleaching (3 sessions, 7 days apart, 30 min/session, irradiated with violet light-LT). Color changes (ΔE_00_, ΔWI_D_), mineral content (CO_3_^2−^, PO_4_^3−^), and topography were assessed (spectrophotometer, ATR-FTIR, and AFM) before and after bleaching procedures (14 days). Metabolic status and three-dimensional components of non-disrupted *Streptococcus* *mutans* biofilms were investigated using a multimode reader and confocal microscopy. The results indicate that ΔE_00_ and ΔWI_D_ significantly increased with NPs’ concentrations and LT. The enamel’s mineral ratio was adversely impacted by HP, but alterations were less pronounced when using NP-containing gels. The enamel’s topography was not damaged by the bleaching protocols tested. The bioluminescence results show that bleaching protocols do not render latent antibacterial properties to enamel, and the confocal microscopy results demonstrate that the 3-dimensional distribution of the components was affected by the protocols. The proposed nanotechnology improved the bleaching efficacy of experimental materials independent of hydrogen peroxide or irradiation and did not adversely impact the enamel’s surface properties or its chemical content.

## 1. Introduction

In-office power bleaching (IPB) is considered an ultraconservative and minimally invasive treatment capable of resolving dental discolorations (low to moderate) in as short as one clinical session [1]. The IPB treatment typically involves three clinical sessions (45 min each; 7 days apart) using hydrogen peroxide-containing bleaching gels (HP, 35% to 45%), in combination with visible light irradiation or not [2], to promote the attainment of immediate esthetic outcomes. IPB’s underlying mechanism of action revolves around the generation of reactive oxygen species (ROS). Upon generation, these short-lived and highly reactive free radicals must be efficiently transported from the gel to the dentin-enamel junction (DEJ). Once at the DEJ, free radicals will then break conjugated double bonds present in large organic molecules (chromophores) through a non-specific oxidative process [3].

Even though several reports have demonstrated the bleaching efficacy of IPB [4,5,6], other studies have indicated that the utilization of these highly caustic bleaching agents may result in the occurrence of adverse effects (short- and long-term), including irreversible changes in enamel topography [7] and chemical make-up [8,9,10], decreased surface microhardness [11], increased surface roughness [12], diminished bond strength [13], and reduced fracture resistance [14]. From the clinical standpoint, the most prevalent adverse effect reported by patients and clinicians is mild to severe dentin hypersensitivity (DH) [4,15,16]. According to previous studies, there is a strong and positive correlation between dentin hypersensitivity, HP concentration, and pulpal cytotoxicity [17,18], where the higher the HP concentration, the stronger the dentin hypersensitivity [16], and the effects are more durable.

In this critical context, several research groups have tried to overcome the limitations described by adding calcium or fluorine ions in the formulation of highly concentrated bleaching gels. Even though the reported results have demonstrated that adverse effects such as decreased enamel microhardness and rougher surfaces were less pronounced with the utilization of calcium- or fluorine-containing gels [8], subsequent studies have shown that the promising results initially reported were limited to the outermost layers of enamel and did not prevent the loss of minerals at subsurface levels, thereby restricting the therapeutic effect of the novel formulations proposed [9]. Follow-up studies investigated the efficacy of experimental protocols modulated by low-concentrated bleaching gels (6–15%) and near-UVA wavelengths (405 ± 15 nm) as an alternative approach to reducing the incidence of dentin hypersensitivity while trying to achieve desirable whitening outcomes [19,20,21]. Even though the utilization of low-concentrated bleaching gels resulted in lower incidences of DH, the bleaching efficacies reported (in terms of ΔE and whitening index (WI)) were considered poor because the outcomes were much less intense and durable as compared to those attained with gels containing high HP concentrations.

Recent approaches focused on the incorporation of metaloxides, such as titanium dioxide (TiO_2_, P25 Degussa) and nitrogen-doped titanium dioxide (N_TiO_2_) nanoparticles, into the formulation of commercially available bleaching gels containing high HP concentrations [22,23,24]. In theory, the incorporation of these semiconductors would improve the dissociation of HP into ROS by a photo-physical process where photons are converted into thermal energy. However, despite the theoretical feasibility of the process, experimental bleaching gels containing varying concentrations of metaloxide nanoparticles were demonstrated to be clinically ineffective when compared to unaltered gels containing HP (either 15% or 35%) [22,23]. These unexpected findings are believed to have precipitated from fast and spontaneous dissociation processes that take place when HP is exposed to metaloxides and from other contributing factors such as limited wettability and high viscosity.

A recent study reported on the successful fabrication of N_TiO_2_ (6–15 nm) using highly controllable, reproducible, and green solvothermal reactions [25]. In that study, synthesized nanoparticles were incorporated into commercially available dental adhesive resins (OptiBond Solo Plus, Kerr Corp., Orange, CA, USA) with the objective of imparting non-leaching antibacterial and biomimetic functionalities to the parental polymer. According to Huo et al. [26], the synthesis route reported by Esteban Florez et al. [25] results in the attainment of pure and crystalline TiO_2_ nanoparticles (anatase phase) that are electron deficient; display high levels of nitrogen doping; have well-defined pore structure, large surface areas, and facilitate the generation of electron-hole pairs; and are capable of efficiently absorbing visible wavelengths (400 to 700 nm) while generating significant amounts of perhydroxyl (HO_2_^•^) and hydroxyl (OH^•^) radicals [25], which are long-lived species of oxygen.

Follow-up studies from the same research group demonstrated the successful solvothermal synthesis of TiO_2_ nanoparticles that were co-doped with either nitrogen and fluorine (NF_TiO_2_) or nitrogen and silver (NAg_TiO_2_), functionalized into OptiBond Solo Plus, and tested for antibacterial properties (in dark and light irradiated conditions) against *Streptococcus mutans* using a newly developed and optimized high-throughput bioluminescence assay [27,28]. According to results reported, experimental materials containing 30% of either NF_TiO_2_ or NAg_TiO_2_ displayed antibacterial behaviors that were comparable to those attained with Clearfil SE Protect (Kuraray Co., Tokyo, Japan; fluoride-releasing material) independently of light irradiation conditions [27]. These findings have not only indicated that the nanotechnology reported has a strong potential to be translated into commercial products capable of sustaining long-term antibacterial properties, but the promising antibacterial effects observed in the absence of light corroborate the findings reported by Huo et al. [26] that nanoparticles synthesized through solvothermal processes are capable of generating long-lived species of oxygen. 

Based on that premise and considering that fluorine is one of the most reactive chemical elements known to man, our research group decided to functionalize NF_TiO_2_ (NP) into experimental bleaching gels containing HP (6%, 15%, or 35%) and determine the effects of nanoparticles’ concentrations (0%, 5%, and 10%) and violet light irradiation on bleaching efficacy, bovine enamel chemical make-up, and surface topography. Additional analyses were focused on revealing how experimental bleaching protocols affect the metabolism and the components of single-species biofilms using a minimally invasive, real-time, and high throughput bioluminescence assay and a concurrent staining technique, along with confocal laser scanning microscopy, respectively. The null hypotheses were that the incorporation of NP would not significantly affect (i) the bleaching efficacy and (ii) the chemical make-up of enamel bleached with the experimental bleaching gels. In addition, it was hypothesized that the incorporation of NP would not (iii) avoid the growth of biofilm on the bleached enamel surface.

## 2. Materials and Methods

### 2.1. Experimental Design

The specimens described in Section 2.2 below (n = 200; n = 10/group) were randomly allocated according to the study factors:

Bleaching Agent:

- 0% hydrogen peroxide (0% HP);
- 6% hydrogen peroxide (6% HP);
- 15% hydrogen peroxide (15% HP);
- 35% hydrogen peroxide (35% HP).

NF_TiO_2_ Concentration (*v/w*):

- 0% NF_TiO_2;_
- 5% NF_TiO_2;_
- 10% NF_TiO_2._

Light Activation:

- Dark conditions;
- Visible light (LT).

Analyses of color (ΔE_00_, ΔWI_D_), pH of the experimental gels, mineral composition (carbonate:phosphate ratio), surface topography of the enamel, and microbiological activity evaluation were conducted. Initial surface analyses were performed before bleaching—baseline (T_0_). Experimental bleaching protocols consisted of three sessions (T_1_ = first bleaching session, T_2_ = second bleaching session, T_3_ = third bleaching session). Analyses following the bleaching protocols were carried out 14 days after (T_4_) the third bleaching session (T_3_). 

### 2.2. Specimen Preparation and Experimental Groups

Squared-shaped specimens (enamel-dentin blocks; area = 36.0 mm^2^, thickness = 3.0 mm) were obtained from the central buccal area of bovine crowns as described in previous studies [10,29]. The blocks were polished using a rotary polisher (Arotec, São Paulo, SP, Brazil) and abrasive disks (600- and 1200-Grit, Norton Saint-Gobain, Guarulhos, SP, Brazil) and finished using polishing cloths (3M Brazil, Sumaré, SP, Brazil) with diamond suspensions (1 μm, 0.50 μm, and 0.25 μm, Erios, São Paulo, SP, Brazil). Prepared specimens were subjected to Knoop microhardness testing (50.0 g load, 5 s/indentation, 3 indentations/specimen, 100 μm apart; Future Tech FM-ARS, Tokyo, Japan) [30]. Specimens (n = 200; 10/group) with standardized microhardness (296.07 kgf/mm^2^ ± 29.60) were randomly distributed and submitted to bleaching with hydrogen peroxide (HP; 0%, 6%, 15%, and 35%) experimental gels, containing NF_TiO_2_ nanoparticles (NP; 0%, 5%, and 10%), and violet light irradiation (LT; with or without):

- G1—No treatment (control group);
- G2—LT;
- G3—HP6;
- G4—HP6 + LT;
- G5—HP15;
- G6—HP15 + LT;
- G7—HP35;
- G8—HP35 + LT;
- G9—HP6 + 5%NP;
- G10—HP6 + 5%NP + LT;
- G11—HP15 + 5%NP;
- G12—HP15 + 5%NP + LT;
- G13—HP35 + 5%NP;
- G14—HP35 + 5%NP + LT;
- G15—HP6 + 10%NP;
- G16—HP6 + 10%NP + LT;
- G17—HP15 + 10%NP;
- G18—HP15 + 10%NP + LT;
- G19—HP35 + 10%NP;
- G20—HP35 + 10%NP + LT.

### 2.3. Nanoparticles’ Synthesis 

A detailed description of the synthesis of NF_TiO_2_ nanoparticles has been reported in previous publications [25,27,28]. A solution of 1.7 g of Ti(OBu)_4_ (Aldrich, St. Louis, MO, USA; 97%), 4.6 g of C_2_H_5_OH (200-proof Decon Labs, King of Prussia, PA, USA), 6.8 g C_18_H_35_NH_2_ (Aldrich; 70%), 7.1 g of C_18_H_34_O_2_ (Aldrich; 90%), and 5% of NH_4_F (based on Ti content; crystalline, ACS, Alfa Aesar, Tewksbury, MA, USA) was prepared and mixed with an ethanol–water solution (4%, 18-Milli-Q; total weight = 13.10 g). The prepared solutions were transparent before mixing; however, the final solution clouded instantaneously after mixing due to hydrolysis and some micelle formation. The final solution was placed into a high-pressure reaction vessel (Borosilicate Glass-lined; Paar Series 4593, Bench Top Reactor System, Moline, IL, USA), reacted (180 °C, 24 h, 15 psi), and stirred via an external shaft coupled to a turbine impeller (280 rpm). At the end of the 24-h cycle, the solution was removed from the reaction vessel and transferred to a 50 mL falcon tube with a certain amount of ethanol (200-proof, Decon Labs). The solution was centrifuged for 15 min at 8000 rpm. This procedure was repeated two additional times using 20 mL of ethanol. 

### 2.4. Polymer Synthesis and Incorporation of NPs

Experimental bleaching gels were formulated in our laboratory by mixing a commercially available hydrophilic polymer (12.5 g, Carbomer 940 NF, Spectrum, Gardena, CA, USA) with an aqueous solution (distilled, 400 mL, pH = 11), containing KOH (60%, 20 mL), using a planetary and orbital stand-alone mixer (1 cycle at 2000 rpm for 2 min and 2 additional cycles at 2500 rpm for 3 min each; Speed Mixer, DAC 400.1 FVZ, FlackTek Inc., Laudrum, SC, USA). Immediately after mixing, the resulting polymer (pH~6) was observed to be transparent and free of any undissolved polymer (white agglomerates). The experimental polymer was then stored in a black container for at least 24 h (refrigerator, 8 °C). 

Two aliquots (1 mL and 2 mL, respectively) of nitrogen and fluorine co-doped titanium dioxide nanoparticles (NF_TiO_2_, ~40 mg/mL) suspended in ethanol (described in Section 2.3 *Nanoparticles Synthesis*) were placed in individual plastic tubes and were centrifuged (8000 rpm, 5 min) in preparation for polymer incorporation procedures. Ethanol-free nanoparticles were then individually mixed into 20 g of the experimental polymer to render gels containing either 5% or 10% NP. Each nanofilled gel was then mixed at 2450 rpm for 20 s (Speed Mixer, DAC 400.1 FVZ, FlackTek Inc., Laudrum, SC, USA). The final gel continued to be transparent and free of visible agglomerates, but its color became pale yellow due to the successful incorporation and dispersion of NP.

### 2.5. Incorporation of Hydrogen Peroxide (H_2_O_2_)

**Figure 1:**
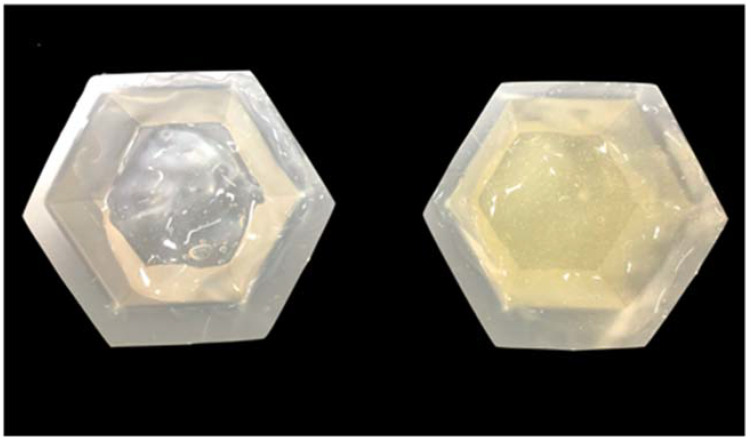
Appearance of experimental polymers without (**left**) or with (**right**) 10% of NP. Note that the incorporation of NPs in the concentration mentioned rendered experimental materials that were transparent a pale-yellow color and free of large agglomerates.

Immediately before utilization, experimental gels (either 1 g or 1.5 g, depending on the H_2_O_2_:polymer ratio) with or without NP (either 5% or 10%) were manually mixed (1:2 (6% or 15% H_2_O_2_) or 2:3 (35% H_2_O_2_)) with 1 mL of hydrogen peroxide following previously published protocols (Figure 1) [10,19]. The rationale for the utilization of two distinct H_2_O_2_:polymer ratios was based on the need to achieve comparable viscosities for all experimental materials investigated.

### 2.6. Bleaching Protocols

**Figure 2:**
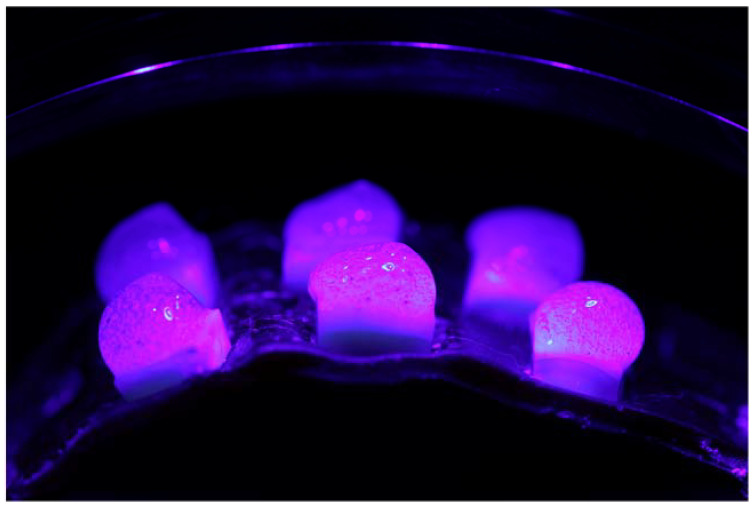
Specimens from G14—HP35 + 5NP + LT being subjected to IPB with visible light irradiation.

The experimental bleaching protocols investigated consisted of 3 sessions (T_1_ = first bleaching session, T_2_ = second bleaching session, and T_3_ = third bleaching session) 7 days apart. Each 30 min session was based on a single application of the proper experimental gel (with or without nanoparticles), combined or not, with continuously visible light irradiation (20 cycles of 1 min, 30 s intervals between irradiation cycles [19]; 405 ± 15 nm, 1.2 W/cm^2^, emission window area = 10.7 cm^2^, Bright Max Whitening, MMO, São Carlos, SP, Brazil) according to experimental groups (G1 to G20; see group descriptions in Section 2.2*. Specimen Preparation and Experimental groups*). Figure 2 illustrates specimens subjected to dental bleaching procedures modulated by experimental bleaching gels and visible light irradiation (G14—HP35 + 5%NP + LT). After each session, specimens from all groups were stored (37 °C, dark conditions) in artificial saliva (1.5 mM calcium chloride [CaCl_2_], 0.9 mM sodium phosphate [NaH_2_PO_4_], and 0.15 mM potassium chloride [KCl, pH 7.0]). After the third session (T_3_), specimens were then stored in artificial saliva for 14 days using the same procedures previously described [10].

### 2.7. Objective Colorimetric Evaluation

The objective colorimetric evaluation (in terms of L*, a*, and b*) was performed before the first bleaching session (baseline, T_0_) and 14 days after the last bleaching session (T_4_) using a hand-held digital spectrophotometer (Vita EasyShade, VITA Zahnfabrik H. Rauter GmbH & Co. KG, Bad Sackingen, Germany). Variation of color (T_4_ − T_0_) was determined using the formulae for ΔE_00_ (Equation (1)) [31,32] and ΔWI_D_ (Equation (2)) [33] as follows:

(1) $$\Delta E_{00}=\sqrt{{\left(\frac{\Delta L^{\prime }}{K_{L}S_{L}}\right)}^{2}+{\left(\frac{\Delta C^{\prime }}{K_{C}S_{C}}\right)}^{2\;}+{\left(\frac{\Delta H^{\prime }}{K_{H}S_{H}}\right)}^{2}+RT\;\cdot \;\left(\frac{\Delta C^{\prime }}{K_{C}S_{C}}\right)\;\cdot \;\left(\frac{\Delta H^{\prime }}{K_{H}S_{H}}\right)\;\;}$$

WI_D_ = 0.55L* − 2.32a* − 1.100b* (2)

### 2.8. pH Analysis

The temporal evolution (10 min increments, total time = 30 min) of pH was determined for experimental bleaching gels (1 g of each, with or without nanoparticles) irradiated or not with visible light using a calibrated pH meter (AB150, Accumet, Fisher-Scientific, Hampton, NH, USA) to determine the impact of pH on the investigated properties. This analysis was carried out during the last bleaching session (T_3_).

### 2.9. Mineral Content Evaluation

Infrared spectra of bovine enamel at T_0_ and T_4_ were acquired at three locations per specimen using a Fourier Transform Infrared spectrometer (Nicolet IS50, Thermo Fisher, Madison, WI, USA; scanning parameters: 500–4500 cm^−1^; resolution 4 cm^−1^, 10 internal scans per spectrum/location) coupled to a heated attenuated total reflectance (ATR) monolithic diamond crystal (Golden Gate, Specac, Fort Washington, PA, USA). A method previously described [7,34] was utilized to guarantee that the ATR-FTIR measurements were performed exactly at the same locations in each specimen. Enamel spectra (at T_0_ and T_4_) from each specimen were corrected for the presence of water before being subjected to baseline correction and normalization procedures using the OMNIC software (v7.0, Madison, WI, USA). The areas under the peaks corresponding to CO_3_^2−^ υ_2_ (886 cm^−1^), PO_4_^3−^ υ_1_ (996 cm^−1^), and PO_4_^3−^ υ_2_ (1410–1460 cm^−1^) were calculated after experimental treatments (T_4_). The mineral composition of enamel (in terms of the carbonate:phosphate mineral ratio) was determined by integrating the areas under the curves of CO_3_^2−^ υ_2_ and PO_4_^3−^ (υ_1_ and υ_2_) [7].

### 2.10. Topography Assessment

An atomic force microscope (MultiMode with Nanoscope V controller, Bruker, Billerica, MA, USA) in ScanAsyst mode coupled with silicon nitride probes (aluminum-coated, triangular, radius = 2 nm, spring constant = 0.4 N/m, Bruker) was used to reveal topographical aspects of specimens (n = 1/group) at T_0_ and T_4_. Images (A = 625 μm^2^; 512 × 512 lines) were acquired (at the same locations at T_0_ and T_4_) using a scan rate of 0.8 Hz. Images were then flattened before acquiring topographical parameters of interest (R_a_ (roughness average) and R_q_ (root mean square roughness)) using the Nanoscope software (v9.0, Bruker). 

### 2.11. Metabolic Status of Non-Disrupted Biofilms

A minimally invasive, real-time, and high throughput bioluminescence assay recently reported by Esteban Florez et al. [27] determined the metabolic status of non-disrupted *Streptococcus mutans* biofilms grown on the surfaces of specimens only after being treated (T_4_) and in accordance with experimental groups (n = 18/group) described in Section 2.2 (*Specimen Preparation and Experimental groups*). These specimens were prepared especially for this methodology. In brief, planktonic cultures of *Streptococcus mutans* (JM10) were grown overnight (16 h) in a liquid culture medium (THY) at oral temperature. Cultures having an optical density higher than 0.900 (at 600 nm; corresponding to 6.43 e^+12^ CFU/mL) were used as inoculum to grow biofilms. *S. mutans* biofilms were then grown (24 h, microaerophilic conditions, 37 °C) on the surfaces of sterile specimens (UV-sterilized, 254 nm, 800,000 μJ/cm^2^, UVP Crosslinker, model CL-1000, UVP, Fisher Scientific, Hampton, NH, USA) using inoculated biofilm growth media (0.65x THY, 1:50 dilution, 1.0 mL/well) supplemented with sucrose (1%, *w/v*). After 24 h, biofilms were immersed in 1.0 mL of a fresh 1x THY + 1% (*w/v*) glucose recharge medium and were incubated (37 °C, 1 h) before being transferred into the wells of sterile white 24-well plates containing 1.0 mL of a fresh 0.65x THY + 1% (*w/v*) sucrose medium. An aqueous solution (100 mM) of D-Luciferin suspended in a citrate buffer (0.1 M, pH 6.0) was added by a Synergy-HT multimode plate reader (Biotek, Winooski, VE, USA) to the wells containing both the specimens and biofilms in a recharge medium (2:1 ratio [*v/v*] inoculum to D-Luciferin). The metabolic activity of non-disrupted biofilms was assessed (in terms of RLUs) at 590 nm in 2 min increments (a total of 6 min) after the addition of D-Luciferin. 

### 2.12. Staining and Confocal Microscopy

A concurrent staining method previously reported by Khajotia et al. [35] was used to illustrate the impact of experimental bleaching treatments on the 3-dimmensional distribution of nucleic acid, proteins, and extracellular polymeric substance (EPS). To achieve this goal, an additional set of specimens (n = 1/group) were prepared and bleached according to the methods previously described (Section 2.2 and Section 2.6). Biofilms were grown on the surfaces of sterile specimens at T_4_ using the methods described in Section 2.11. After the 24 h growth period, biofilms were washed with PBS (3x, pH 7.4, 25 °C, 15 s/wash) to remove non-adherent cells. Biofilms were then concurrently stained with Alexa Fluor^®^ 647 conjugate of Concanavalin A (Invitrogen, Waltham, MA, USA; 250 μg/mL), Syto 9 (Molecular Probes, Eugene, OR, USA; 10 μM), and Sypro Red (Invitrogen, USA; 10x). Biofilms were kept hydrated in sterile, ultra-pure water and protected from light until confocal microscopy. Images of biofilms were acquired using a TCS-SP2 MP confocal laser scanning microscope (CLSM, Leica Microsystems, Inc., Buffalo Grove, IL, USA) with Ar (488 nm) and He/Ne (543 and 633 nm) lasers for the excitation of the fluorescent stains within biofilms at three different locations on each specimen’s enamel surface. A 63x water immersion microscope objective lens was used. Serial optical sections were recorded from the surface of specimens to the top of biofilms at 0.6 μm intervals on the Z-axis. Three-dimensional images of the biofilms were generated using Volocity software (PerkinElmer, Waltham, MA, USA) to allow the visualization of the distribution of the nucleic acid (green fluorescence), proteins (red fluorescence), and EPS (blue fluorescence) components of biofilms.

### 2.13. Statistical Analyses

Linear Models (two- and three-way ANOVA) with outcomes including ΔE_00_ and ΔWI_D_ (T_4_ − T_0_) and the carbonate:phosphate mineral ratio (only at T_4_) were fitted. The mineral ratio data were transformed into log. Factors included HP concentration (4 levels: without HP, 6% HP, 15% HP, and 35% HP), NP concentration (3 levels: without NP, 5% NP, and 10% NP), and LT (2 levels: with or without light). A backward model selection strategy was adopted with the full model containing all the main effects, all the two-way interactions (HP*NP, HP*LT, NP*LT), and the three-way interaction (HP*NP*light). A term was removed from the model if its *p*-value was less than 0.05 and the removal process started with the highest order term. In addition, RLU obtained from the metabolic status analysis of the biofilms were submitted to the general linear models test considering the group (G1–G20) and time (0, 2, 4, and 6 min) with post hoc Student–Newman–Keuls tests. All the analyses were conducted using SAS software (version 9.3, SAS Institute, Cary, NC, USA) at a 5% level of significance.

## 3. Results

### 3.1. Bleaching Efficacy

**Figure 3:**
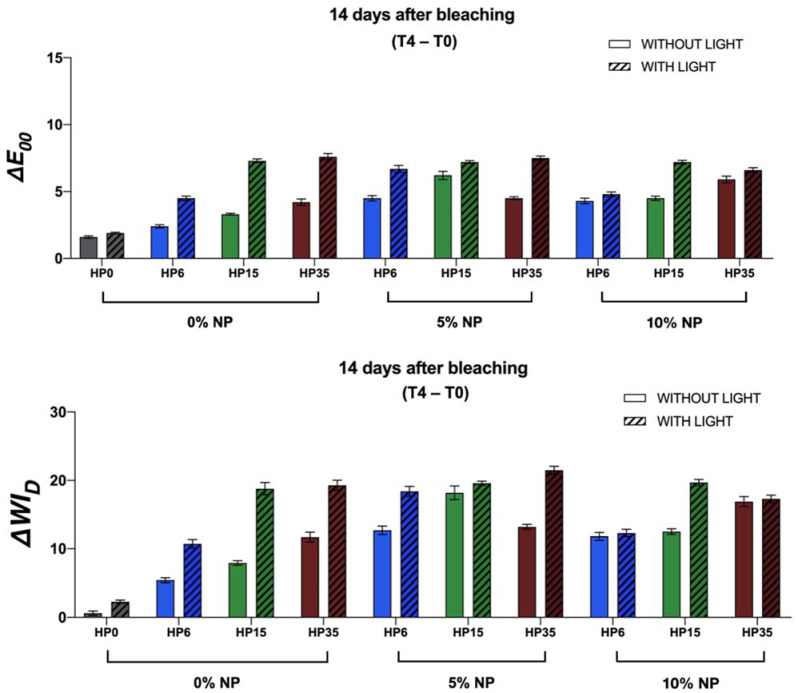
Mean and standard error values of ΔE_00_ and ΔWI_D_ that were calculated considering the coordinate values collected before (T_0_) and 14 days (T_4_) after the last bleaching session with 6%, 15%, and 35% HP incorporated or not with NP (either 5 or 10%).

**Figure 4:**
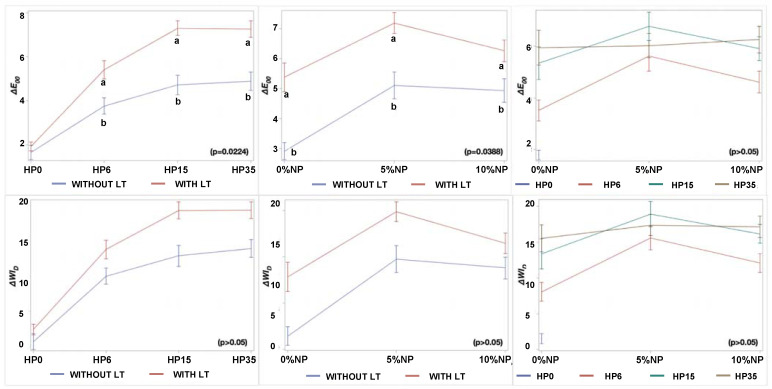
Marginal ΔE_00_ and ΔWI_D_ means and standard errors within each level of HP (0%, 6%, 15%, 35%), NP (0%, 5%, and 10%), and LT (with or without) according to two-way interactions, HP*LT, NP*LT, and NP*HP, respectively. Distinct letters represent a difference within the same HP or NP concentrations, taking into consideration a 0.05 level of significance.

The findings reported in Figure 3 have demonstrated that experimental bleaching protocols modulated by gels containing 6%, 15%, and 35% H_2_O_2_ (without NP or LT) displayed mean values of ΔE_00_ and ΔWI_D_ that were higher when compared to those of the control groups (no treatment, with, or without LT). The two-way interactions among the factors are displayed in Figure 4. These results have also indicated that the ΔE_00_ and ΔWI_D_ values varied with HP concentrations (*p* < 0.0001), and bleaching outcomes could be ordered in terms of increasing efficacies where HP6 < HP15 < HP35, respectively. Even though a similar trend was observed when bleaching protocols were modulated by experimental bleaching gels containing 6%, 15%, and 35% HP (without NP) and visible light irradiation (405 nm ± 15 nm), the ΔE_00_ and ΔWI_D_ values were higher than those from bleaching protocols with no light irradiation (*p* < 0.0001). The combination of HP and NP further increased the efficacy of experimental bleaching protocols, as denoted by mean ΔE_00_ and ΔWI_D_ values of HP6 and HP15 incorporated with 5% NPs.

### 3.2. Analysis of pH

**Figure 5:**
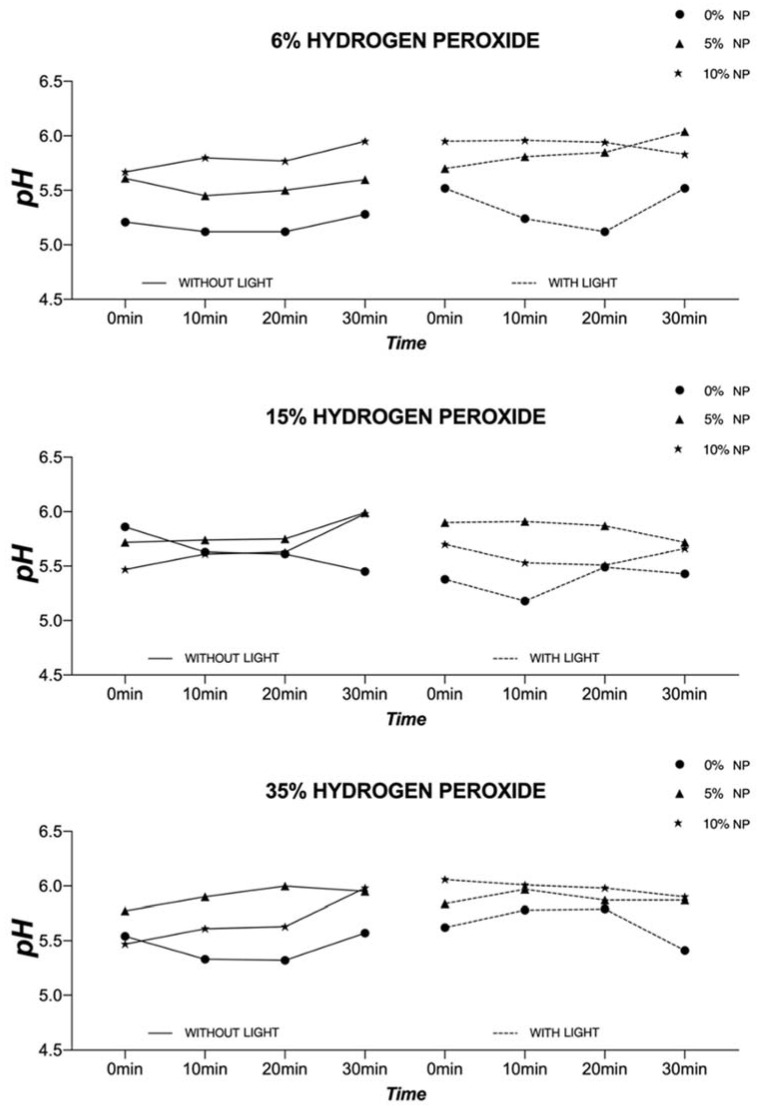
Temporal evolution (0, 10, 20, and 30 min) of pH values of the experimental gels (6%, 15%, and 35% HP) with or without NP and LT.

The graphs displayed in Figure 5 illustrate the temporal evolution of pH for experimental gels (6%, 15%, and 35% HP) with or without NP (5% and 10%). It is possible to observe that experimental gels (6%, 15%, and 35% HP), without the incorporation of NP, displayed pH values (≅5.0, 6% HP + LT at 20 min) that were lower when compared to those containing NP. Such behavior was observed to be consistent throughout the observation time (at 0, 10, 20, and 30 min). The results reported have also indicated that such behavior is not influenced by visible light irradiation and that nanofilled bleaching gels displayed comparable pH values after 30 min. 

### 3.3. Mineral Content Evaluation

**Figure 6:**
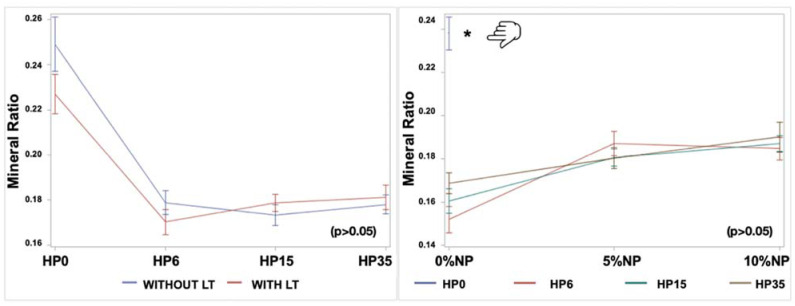
Carbonate:phosphate mineral ratio marginal means and standard errors within each level of HP (0%, 6%, 15%, 35%), NP (0%, 5% and 10%), and LT (with or without) according to the two-way interactions, HP*LT and NP*HP, respectively. The finger icon points to the control group (HP0), and the asterisk represents the difference between the control and the other groups, taking into consideration a 0.05 level of significance.

**Figure 7:**
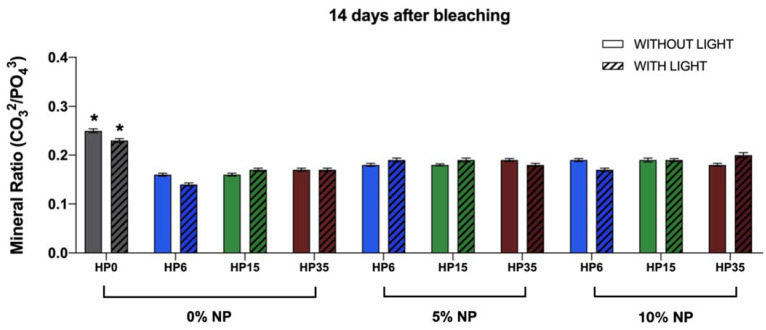
Mean and standard error values of the mineral ratio acquired after 14 days from bleaching (T_4_) from the integrated areas of CO_3_^2−^ υ_2_ to PO_4_^3−^ υ_1_, υ_2_ contours. The asterisks represent the difference between the control groups and the other ones, taking into consideration a 0.05 level of significance.

The linear models showed that only the isolated factors, HP (*p* < 0.0001) and NP (*p* < 0.0001), were significant to the carbonate:phosphate mineral ratio variable. The LT factor and the two-way and three-way interactions were not significant (Figure 6). Figure 7 illustrates the impact of experimental bleaching gels on the mineral ratio CO_3_^2−^/PO_4_^3−^ of bovine enamel. It is possible to observe a decrease of T_4_ in all groups investigated where values varied from 0.14 ± 0.03 (6HP + LT) to 0.20 ± 0.05 (35HP + NP10% + LT) compared to the control groups (G1 and G2). 

**Figure 8:**
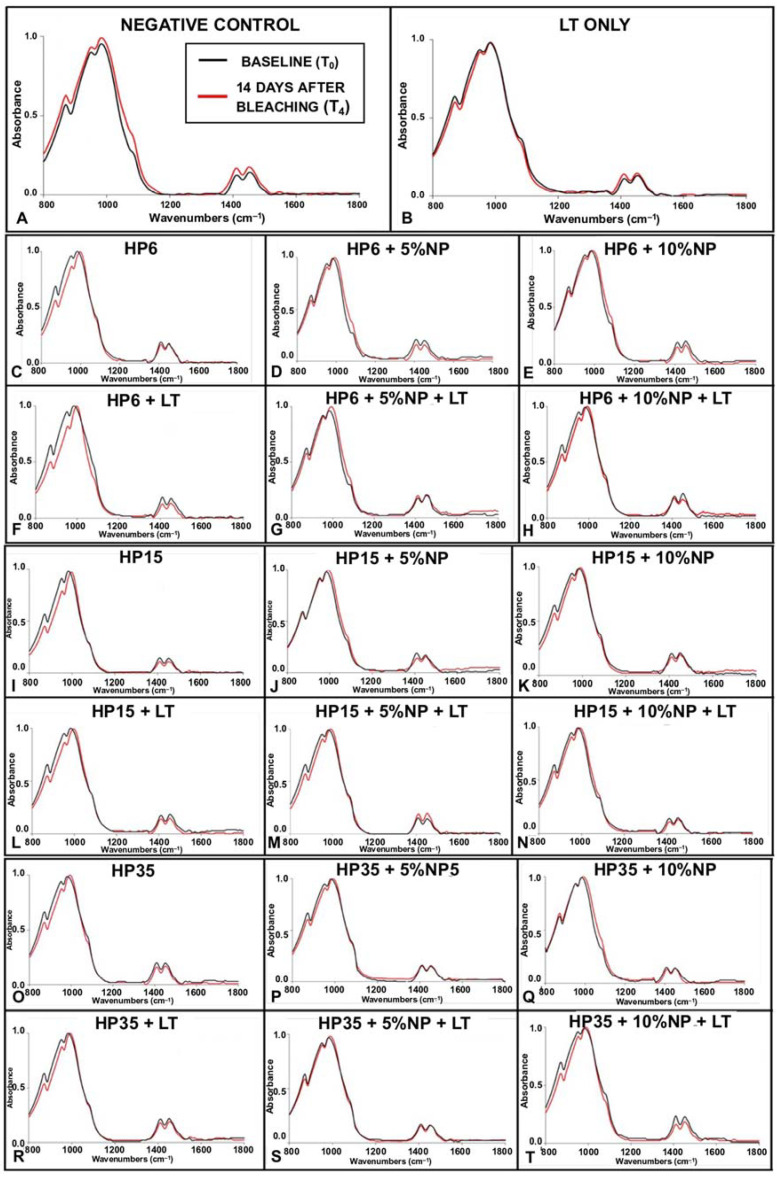
(**A**–**T**) ATR-FTIR spectra of specimens before (black curves, (T_0_)) and 14 days after (red curves, (T_4_)) being treated with experimental bleaching gels containing HP (6%, 15%, and 35%), with or without NF_TiO_2,_ and with or without visible light irradiation (LT).

Figure 8A–T illustrates the results from the ATR-FTIR analysis of the mineral content of enamel before (baseline (T_0_), black curves) and after bleaching (14 days after (T_4_), red curves). Figure 8A indicates (in terms of normalized absorbance values) that specimens pertaining to G1 (negative control) displayed spectra characterized by absorbance values that were slightly higher at T_4_ (wavenumbers from 800 cm^−1^ to 1150 cm^−1^ and from 1350 cm^−1^ to 1550 cm^−1^) and by a larger spectral bandwidth at absorbance values between 0.0 and 0.5. In combination, these results indicate that the mineral content of enamel was not altered by storing specimens in artificial saliva for the duration of the experiment. Similar behavior was observed in Figure 8B, which demonstrate that the utilization of violet light irradiation (in the conditions tested) also did not promote any changes to the chemical composition of treated enamel. 

Figure 8 (C, I, and O; HP (6%, 15%, and 35%)), (F, L, and R; HP (6%, 15%, and 35%) + LT), (D, J, and P; HP (6%, 15%, and 35%) + NP (5%)), (G, M, and S; HP (6%, 15%, and 35%) + NP (5%) + LT), (E, K, and O; HP (6%, 15%, and 35%) + NP (10%)), and (H, N, and T; (6%, 15%, and 35%) + NP (10%) + LT) illustrate the results for specimens that were subjected to experimental bleaching protocols. It is possible to observe that specimens treated with HP (either 6%, 15%, and 35%), with or without LT (Figure 8C,F,I,L,O,R), displayed spectra at T_4_ that were characterized by lower absorbance values for the CO_3_^2−^ υ_2_ (886 cm^−1^) and PO_4_^3−^ υ_1_ (996 cm^−1^) peaks. In addition, it was possible to observe that the combination of HP and LT shifted the spectra to the right (wavenumbers between 800 cm^−1^ and 1150 cm^−1^). Such behavior was more drastic for specimens treated with either 6% or 15% HP and LT (Figure 8F,L). This behavior was not observed in specimens treated with bleaching gels containing NP independent of light irradiation (with or without) or nanoparticles’ concentrations (either 5% or 10%). In these instances, spectra were observed to display shapes and absorbance values that were similar to those of the control group (no treatment, stored in artificial saliva) at T_4_.

### 3.4. Topography Assessment

**Figure 9:**
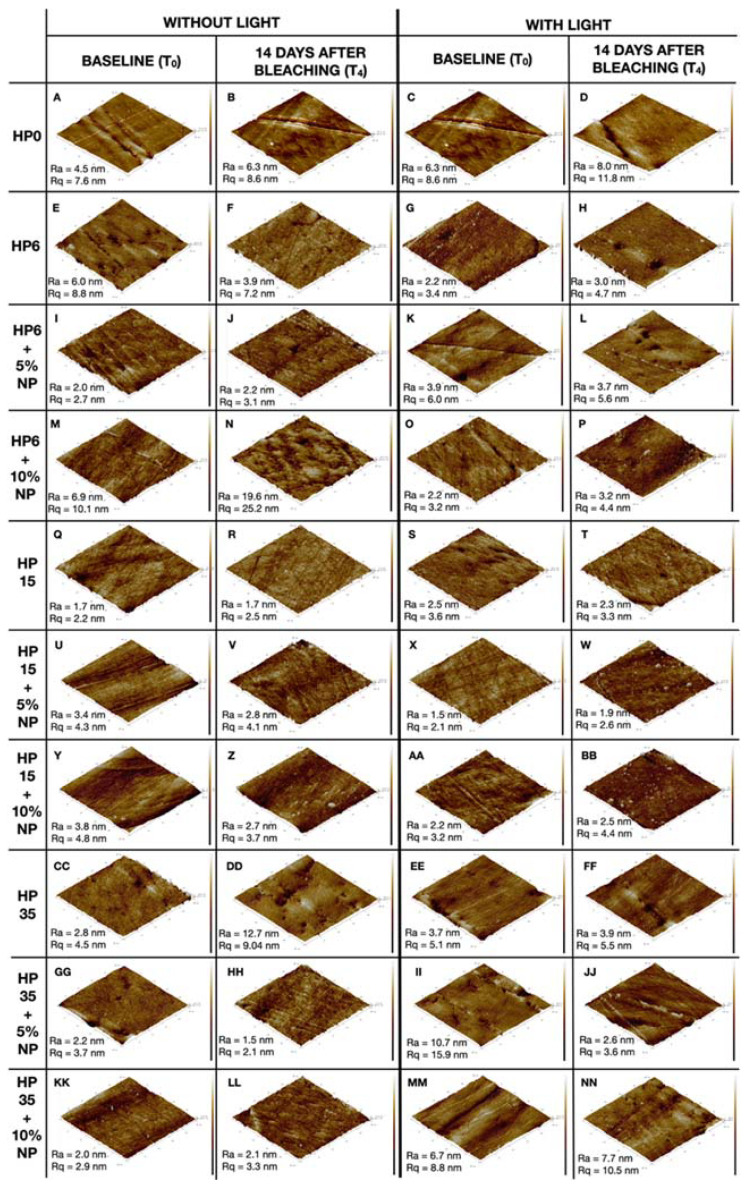
(**A**–**NN**) Images acquired using atomic force microscopy showing illustrative areas of the enamel before (T_0_) and after 14 days (T_4_) from bleaching with HP6, HP15, and HP35, with corresponding %NF_TiO_2,_ and the presence or absence of LT. Control groups are represented by HP0. Ra and Rq displayed in each subfigure indicate roughness average and root mean square roughness, respectively.

Illustrative results from the topographical assessment performed with AFM are shown in Figure 9A–NN where it is possible to observe that R_a_ and R_q_ values varied from 1.5 nm (HP35 + 5%NP at T_4_) to 19.6 nm (HP6 + 10%NP) and from 2.1 nm (HP35 + 5%NP) to 25.2 nm (HP6 + 10%NP), respectively. It is possible to observe through the images that, in most cases, the topography of the surfaces was maintained between T_0_ and T_4_. Overall, the surfaces were smooth and few of them illustrated the distribution and direction of enamel prisms. 

### 3.5. Metabolic Status of Non-Disrupted Biofilms

**Figure 10:**
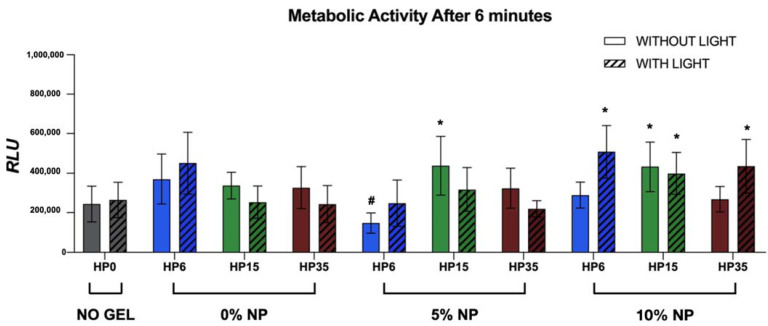
Mean and standard deviations of RLU values 6 min after the addition of D-Luciferin substrate to 24-h *Streptococcus Mutans* (JM10) biofilms. *S. mutans* biofilms were grown on the enamel of individual specimens 14 days after the last bleaching session (T_4_). The hashtag sign denotes groups with RLU values that were statistically lower than those from the control group (no treatment-G1), while asterisks represent groups with RLU values that were statistically higher than those from G1.

The general linear model evaluation detected significance for both the isolated factors of group and time (*p* < 0.0001), but no interaction was reported among them (group*time, *p* = 1.000). Figure 10 illustrates the results, in terms of relative luminescence units (RLU) (mean and standard deviation values 6 min after the addition of D-Luciferin), of the metabolic status of non-disrupted *S. mutans* biofilms grown for 24 h on the surface of the specimens that were previously bleached. It is possible to observe that, except for G9 (HP6 + 5%NP), biofilms grown on specimens treated with experimental bleaching protocols modulated by gels containing HP (6%, 15%, and 35%), with (5% and 10%) or without NP and LT, displayed metabolic statuses that were either comparable to or higher than those observed on G1 (negative control). 

### 3.6. Confocal Microscopy

**Figure 11:**
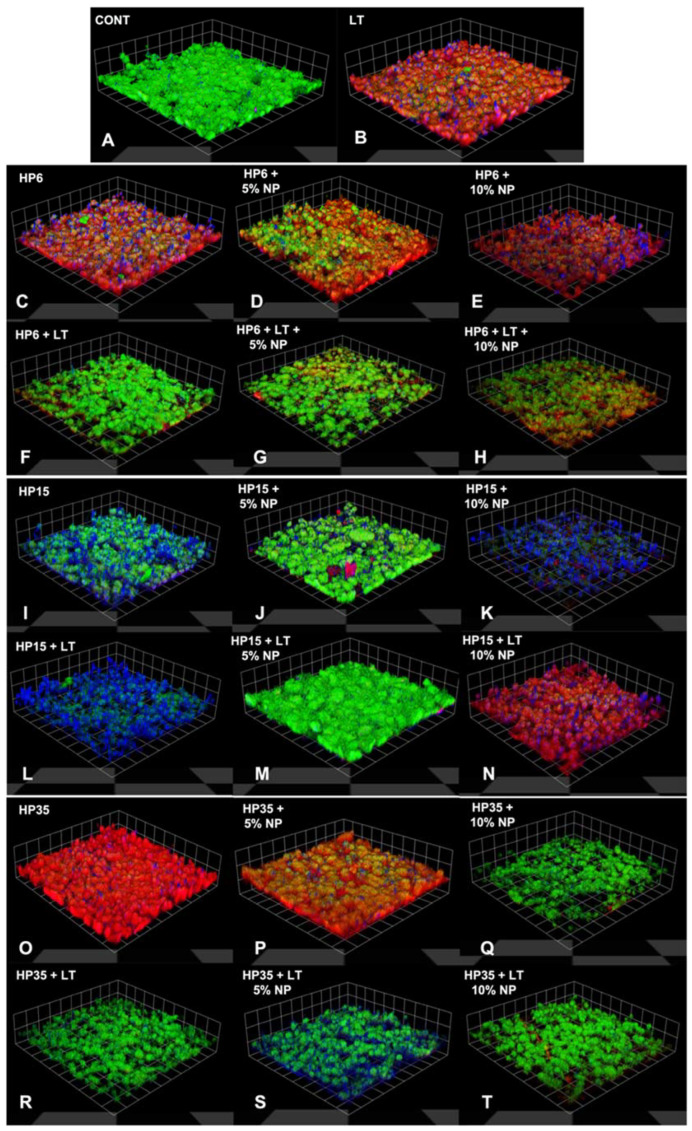
(**A**–**T**) Three-dimensional reconstructions of the biofilms with concurrent staining observed using confocal laser scanning microscopy 14 days after (T_4_) bleaching with 6%, 15%, and 35% HP, with or without the incorporation of 5% and 10% NP. It is possible to detect the distribution of the nucleic acid (green fluorescence), proteins (red fluorescence), and EPS (blue fluorescence) components of biofilms. Biofilms grown on surfaces bleached without LT were more porous, exhibiting mostly red and blue fluorescence signals. A clear trend, in terms of fluorescence signals (either green, red, or blue), could not be observed for specimens treated with experimental bleaching gels containing HP (6%, 15%, or 35%) with (5% and 10%) or without NP and LT.

The results from the concurrent staining and confocal laser scanning microscopy analysis are shown in Figure 11A–R as 3D reconstructions of biofilms where it is possible to observe green (nucleic acids), red (proteins), and blue (EPS) fluorescence channels, the impact of experimental bleaching treatments on components of biofilms, and their three-dimensional distributions. It was possible to detect in Figure 11A that biofilms expressed mostly green fluorescence when grown against the surfaces of specimens that were not treated with experimental bleaching gels (G1—negative control). It was possible to observe that biofilms grown on groups that were not light-irradiated surfaces were not only more porous but also displayed fluorescence signals that were mostly red and blue, which indicates that biofilms were expressing proteins and EPS more intensely. A clear trend, in terms of fluorescence signals (either green, red, or blue), could not be observed for specimens treated with experimental bleaching gels containing HP (6%, 15%, or 35%) with (5% and 10%) or without NP and LT.

## 4. Discussion

Even though the efficacy of IPB [31] has been previously demonstrated by numerous research groups, post-treatment DH continues to be the most frequently reported adverse effect [15,16,17]. In fact, a previous study [36] investigating the correlation between the bleaching efficacy and risk/intensity of post-treatment DH has indicated, based on a multi-regression and logistic analysis, that the risk for the occurrence of DH was 120% more likely to precipitate from IPB than from at-home bleaching techniques [36]. In addition, the intensity of painful symptoms was reported to be at least four times stronger for patients treated with IPB than those subjected to at-home treatments.

According to previous studies, the intensity (low, mild, and severe) and duration (short-term or long-term) of DH precipitate directly from the peroxide concentrations and exposure times used [17]. Therefore, the behavior reported [36] is expected because at-home bleaching gels are three-to-six times less concentrated than those used in IPB [1,2,3], and were demonstrated to be less cytotoxic and to penetrate less into the tooth structure [6,17], thereby diminishing potential risks associated with the vitality of pulpal tissues. Despite these promising results, at-home techniques require long exposure times and result in bleaching outcomes that are only similar to those achieved with IPB [37]. This critical scenario underscores the need for the development of techniques and products that are capable of resolving dental discolorations in a short period of time without causing DH or negatively impacting the properties of teeth (surface, mechanical, and biological).

The present research represents an effort to overcome the limitations cited by developing experimental bleaching gels containing low concentrations of HP, and third-generation titanium dioxide nanoparticles co-doped with nitrogen and fluorine, which have been demonstrated to have non-leaching antibacterial properties [25,27]. Results reported in the present study have demonstrated that the incorporation of NP (5% and 10%, *w/v*) into experimental bleaching gels containing low concentrations of HP (6% and 15%) rendered esthetic outcomes (in terms of ΔE_00_ and ΔWI_D_) that were similar to those attained with high-concentrated bleaching gels (HP35), thereby suggesting that the nanotechnology proposed has the potential to resolve mild-to-severe dental discolorations in short periods of time (3 sessions, 30 min/session) and with a lower amount of hydrogen peroxide. Therefore, the first null hypothesis that NP incorporation would not significantly influence the efficacy of experimental bleaching gels was rejected. The utilization of violet light irradiation (LT; 405 nm ± 15 nm) was shown to improve the efficacy of experimental bleaching gels containing varying concentrations of HP (6%, 15%, and 35%) with or without NP (5% and 10%), as denoted by the statistical outcomes and the mean values of ΔE_00_ and ΔWI_D_ that were higher than those from experimental bleaching gels (with or without NP) in dark conditions, demonstrating that LT is still fundamentally important to achieving good esthetic outcomes.

Tano et al. [38], while investigating the effects of visible light irradiation (405 nm) emitted from a laser source on the efficacy of HP35 + TiO_2_ (0.1% wt/wt) against a methylene blue solution (MB; 1.0 g, 100 ppm in 7.0 g of water), have demonstrated that even small concentrations of HP and TiO_2_ can be efficiently used to bleach organic dyes when in the presence of visible light, thereby further supporting the necessity of using light irradiation and the present study’s rationale for the selection of concentrations investigated (HP and NP).

Despite these promising in vitro results, findings from randomized clinical trials (RCTs) investigating the efficacy of HP6 with either TiO_2_ [39] or N_TiO_2_ [22,40] were less encouraging and have shown that experimental bleaching gels tested [22,39,40] produced less DH but were much less effective (in terms of bleaching outcomes) when compared to commercially available products (35% HP). These findings could have precipitated from the combination between the wavelength selected (450 nm ± 15 nm; 2.76 μeV) [22,40] and the bleaching gels containing nanoparticles fabricated by calcination strategies that behave as semiconductors and cannot generate ROS. In combination, the factors cited result in sub-optimal bleaching reactions and poor esthetic outcomes.

The experimental design of the present study was based on the utilization of shorter wavelengths (405 nm ± 15 nm) with higher photon energies (3.06 μeV) and nanoparticles (NF_TiO_2_; 6–15 nm) that were synthesized using robust and green solvothermal reactions as previously reported by our group [25,27,28]. Nanoparticles reported herein were shown to have well-defined pore-size distributions, be electron deficient, be capable of producing substantial amounts of ROS [26], and result in experimental materials that are more efficient and less aggressive to the tooth structure. A previous study [25] demonstrated that single-doped nanoparticles (N_TIO_2_), fabricated using similar synthetic routes, were capable of absorbing twice as much light (between 200 nm–800 nm) as compared to commercially available nano-TiO_2_ (P25, Degussa) [25]. Since LT has been demonstrated by the present study to be fundamentally important for the success of IPB and, taking into consideration that the reported nanoparticles have the ability to intensely absorb visible wavelengths, it can be hypothesized that the investigated experimental protocols could result in good esthetic outcomes.

This hypothesis has been corroborated by findings reported in a recent randomized, controlled, and double-blind clinical trial [41] that investigated the clinical performance (in terms of immediate ΔE_ab_) of 6% HP with N_TiO_2_ when activated by two distinct wavelengths (405 ± 15 nm and 450 ± 15 nm). The results of that study [41] show that the clinical color change of experimental bleaching gels was less pronounced when using blue irradiation (450 ± 15 nm). The authors have also reported that bleaching protocols modulated by violet radiation displayed bleaching efficacies that were comparable to the control group (35% of HP) but were associated with lower levels of DH. Even though the trends observed [41] validate the results of the present study, it is important to underscore that our experimental design was based on the utilization of the CIEDE2000 formula (ΔE_00_) [32] and the whiteness index (ΔWI_D_), which are considered more relevant for dentistry and dental bleaching investigations.

According to Paravina et al. [42], the mean ΔE_00_ values reported for G10 (HP6 + NP5 + LT) are considered excellent and indicate that experimental protocols modulated by LT and 5% NP resulted in bleaching efficacies that were comparable to those from HP15 (G6, G11, G12) and HP35 (G8, G14, G19, G20). These findings could have precipitated from the spontaneous dissociation that hydrogen peroxide undergoes when in the presence of metaloxides such as NF_TiO_2_ and suggest that experimental bleaching gels containing low concentrations of NP and HP may result in materials with promising bleaching performances. The higher mean values of ΔE_00_ and ΔWI_D_ detected in G11 (HP15 + NP5) serve as additional evidence of such a spontaneous dissociation process. This was expected behavior and has been corroborated by previous research from our group that demonstrated that photocatalysts of similar compositions displayed promising antibacterial properties against *Streptococcus mutans* even in the absence of light irradiation [27].

With regard to the temporal evolution of pH, it was possible to observe that the incorporation of NP (5% and 10%) into experimental bleaching gels containing HP (6%, 15%, and 35%) did not adversely impact the values observed. For experimental gels containing 6% HP, the NP incorporation (either 5% or 10%) resulted in pH values that were higher when compared to gels without NP (with or without LT). Gels containing either 15% or 35% of HP, displayed pH values that fluctuated a bit more, but overall, the incorporation of NP seemed to have a stabilizing effect that prevented the acidification of experimental materials during the investigated period of time (30 min). Our findings contradict the results published by Pretel et al. [43], because, even though commercial gels modified by the incorporation of N_TiO_2_ (produced by calcination strategies) displayed initial pH values that were high, the materials investigated [43] displayed pH values that were significantly lower after 30 min of observation. On the other hand, Monteiro et al. [44] have shown that the incorporation of 1% TiO_2_ nanotubes did not adversely impact the pH values of gels containing either 10% carbamide peroxide (pH ≅ 6.5) or 40% HP (pH ≅ 7.0). These differences in pH values reported in the literature and by the present study (initial and after 30 min) can be explained by the intrinsic differences in materials’ compositions including the type of polymeric matrix and stabilizing agents.

A recent study [43] investigating temporal variations of pH and the electric potential (EP) of three commercially available bleaching gels has demonstrated that there is a strong and positive correlation between pH and EP values, where EP was inversely varied with the evolution of pH. Gentil de Moor et al. [45] have indicated that ROS such as oxygen, hydroperoxyl, sodium hypochlorite, hydrogen peroxide, ozone, and hydroxyl have redox potentials of +1.229 V, +1.510 V, +1.630 V, +1.780 V, +2.075, and +2.800 V, respectively, and therefore, the bleaching gels proposed in the present study are hypothesized to preferentially generate oxygen and hydroperoxyl and to prevent the etching of treated enamel.

In our study, a significant decrease in the mineral ratio was detected for all bleached groups in comparison to the control ones independent of the NP incorporation. Therefore, the second null hypothesis that the NPs would not negatively influence the chemical content of enamel bleached with the experimental gels was rejected. However, the results of the present study suggest a trend of increasing the mineral ratio of enamel bleached with gels containing NP (5% and 10%), which should be further confirmed with additional mechanical and surface testing. Xu et al. [46] already demonstrated the presence of a strong and inverse relationship between the carbonate:phosphate ratio and surface properties of enamel (elastic modulus and hardness). Even though the location of the ATR-FTIR evaluation over time (T_0_ and T_4_) was standardized, the specimens were stored in saliva for 14 days after bleaching as an additional attempt to mimic, as much as possible, the clinical condition [9,10]. As it has already been demonstrated, the presence of saliva may either uphold or recover the levels of mineralization of enamel after tooth bleaching [47].

Another valuable piece of information provided by the ATR-FTIR assessment was the spectra of enamel, demonstrating that experimental bleaching gels containing HP (6%, 15%, and 35%) but without NP (which are more acidic) have negatively impacted the chemical make-up of treated enamel independently of LT, as denoted by a right-shift of the spectrum between wavenumbers between 800 cm^−1^ and 1150 cm^−1^ and lower absorbance values for CO_3_^2−^ υ_2_ (886 cm^−1^) and PO_4_^3−^ υ_1_ (996 cm^−1^). As demonstrated in Figure 5, this behavior was overall not observed in specimens treated with experimental bleaching gels containing NP (either 5% or 10%). In these instances, spectra were observed to have shapes and absorbance values that were similar to those of the control group (no treatment). Although some authors have reported distortions in the peaks mentioned independently of pH values [46,48], Sun et al. [7] have also demonstrated that acidic bleaching agents (30% HP, pH ≅ 3.6) not only decreased CO_3_^2−^ υ_2_ (886 cm^−1^) and PO_4_^3−^ υ_2_ (1410–1460 cm^−1^) absorbance values but have also right-shifted the spectra (between 800 cm^−1^ and 1150 cm^−1^) of treated enamel, thereby further corroborating the findings of the present study. In their study [7], the decrease in the mineral content (CO_3_^2−^/PO_4_^3−^) was followed by significant reductions in the enamel’s surface microhardness. This concerning trend was not detected when the authors used experimental bleaching agents with a neutral pH [7].

It is important to highlight that the evaluation of the carbonate (CO_3_^2−^) content provides valuable information to the field because CO_3_^2−^ represents 2.5% of the weight of the enamel’s composition, behaves as a substitute anion for phosphate or hydroxyl groups in hydroxyapatite [45] (HAp [Ca_10_(PO_4_)_6_(OH)_2_]), and is not stoichiometrically distributed in dental enamel [46]. In addition, the carbonate’s position (at hydroxyapatite’s lattice or surface) not only modifies its shape and size but, more importantly, increases the solubility of carbonated apatites [49,50]. This physico-chemical property of hydroxyapatite could be used to explain the reason why experimental bleaching gels without nanoparticles significantly decreased the mineral ratio of treated enamel. We hypothesize that the trend of the increasing mineral ratio observed when specimens were treated with gels containing NP (5% and 10%) could be explained by the presence of fluorine ions in the crystal lattice of NF_TiO_2_ that could potentially alter the composition of hydroxyapatite (into fluorapatite) through an ion-exchange mechanism. Even though the mechanism is not energetically favorable, the formation of fluorapatite could be responsible for the maintenance of the chemical make-up observed once fluorapatite displays solubility levels that are lower than those of carbonated apatites when exposed to acidic pH values [49,50].

Even though large variations in R_a_ and R_q_ values could be correlated with intense damage to the enamel, the vast majority of our results have indicated that treatments conducted with or without LT, and modulated by experimental gels (6%, 15%, and 35%) with (5% and 10%) or without NP, were not able to adversely impact the surface topography of treated enamel at T_4_, and therefore, the results from the group treated with 6HP+10%NP were considered to not follow the overall trend observed. Additional analyses based on optical profilometry will include a quantitative evaluation of enamel’s surface roughness. Contrary to these findings, others [7,12] have shown that enamel surface properties were negatively impacted by bleaching protocols modulated by high-concentrated bleaching gels (either 30% or 40% HP), independently of their pH values (either acidic or neutral), using similar AFM techniques. Therefore, it could be assumed that the compositions tested have the potential to resolve mild-to-severe dental discolorations without negatively impacting the chemical arrangement or surface properties of treated enamel.

These findings are also important from the oral microbiology standpoint because it is well known that surfaces (either biotic or abiotic) with high mean R_a_ values accumulate more biofilms [51], due to increased surface area and surface energy, and may alter the ecology of biofilms from a state of health into a disease-associated state. The real-time and high throughput bioluminescence assay performed in the present study had the objective of determining (i) if treated enamel surfaces would display a latent antibacterial behavior and (ii) if treated surfaces would support more biofilm growth. As demonstrated by the results shown in Figure 10, experimental bleaching treatments modulated by HP (6%, 15%, and 35%) and NP (5% and 10%) were not capable of rendering antibacterial effects to treated surfaces against non-disrupted biofilms of *Streptococcus mutans* independently of LT, as denoted by RLU values that were either similar or higher to those from the control group (no treatment). Therefore, the last null hypothesis that the incorporation of NP would not avoid the growth of biofilm after bleaching was accepted. In the past, Ittatiruti et al. [51] demonstrated that, by using the viable colony counting assay (CFU/mL), dental bleaching procedures modulated by bleaching gels containing either 25% or 35% of HP did not promote higher accumulations of *S. Mutans* but increased the *S. Sanguinis* biofilm formation.

The only exception to the trend observed in our work was in specimens from G9 (HP6 + NP5) that displayed biofilms with RLU values that were slightly lower than those from the control group (no treatment). These findings suggest that enamel surfaces treated with HP6 + 5%NP could potentially become antibacterial by the utilization of the nanotechnology proposed, but additional studies are necessary to confirm these findings and elucidate potential mechanisms of action associated. The qualitative results from the confocal microscopy analysis (Figure 11) have indicated that enamel surfaces treated with experimental bleaching protocols modulated by HP (6%, 15%, and 35%) and NP (5% and 10%) with or without LT, promoted the growth of biofilms displaying different three-dimensional distributions of nucleic acids, proteins, and EPS, thereby suggesting that bleached enamel may indeed impact the accumulation and growth of oral biofilms. However, these results should be interpreted with caution because the qualitative data reported cannot be considered representative due to the small number of specimens analyzed.

Subsequent studies from our group will investigate the mechanisms of action by which the proposed nanoparticles (i) improve the efficacy of experimental bleaching gels, (ii) maintain the mineral content of treated enamel, and (iii) modulate the three-dimensional distribution of biofilms’ components. Our group is also planning the execution of a controlled, randomized, and double-blind clinical trial to determine the clinical efficacy of experimental bleaching gels proposed herein.

## 5. Conclusions

The present study has successfully demonstrated the synthesis of experimental bleaching gels using hydrophilic polymers and functionalized nanoparticles. The nanotechnology reported was demonstrated to significantly improve the bleaching efficacy of experimental materials independent of hydrogen peroxide or light irradiation and did not adversely impact the surface properties or chemical make-up of treated enamel. The results of the present study have shown that experimental materials were not capable of rendering antibacterial effects to treated enamel but were observed to alter the three-dimensional distribution of components within *S. mutans* biofilms. Subsequent studies should not only investigate the mechanisms of action by which the proposed nanotechnology improves bleaching reactions but also demonstrate the clinical efficacy of the proposed materials.

## Data Availability

Not applicable.
